# Retrospective on the First Passive Brain-Computer Interface Competition on Cross-Session Workload Estimation

**DOI:** 10.3389/fnrgo.2022.838342

**Published:** 2022-04-04

**Authors:** Raphaëlle N. Roy, Marcel F. Hinss, Ludovic Darmet, Simon Ladouce, Emilie S. Jahanpour, Bertille Somon, Xiaoqi Xu, Nicolas Drougard, Frédéric Dehais, Fabien Lotte

**Affiliations:** ^1^ISAE-SUPAERO, Université de Toulouse, Toulouse, France; ^2^Artificial and Natural Intelligence Toulouse Institute ANITI, Toulouse, France; ^3^Inria Bordeaux Sud-Ouest, Talence, France; ^4^LaBRI (CNRS, Univ. Bordeaux, INP), Bordeaux, France

**Keywords:** benchmarking, dataset, passive brain-computer interface, workload, EEG, cross-session variability, estimation, Riemannian geometry

## Abstract

As is the case in several research domains, data sharing is still scarce in the field of Brain-Computer Interfaces (BCI), and particularly in that of passive BCIs—*i.e*., systems that enable implicit interaction or task adaptation based on a user's mental state(s) estimated from brain measures. Moreover, research in this field is currently hindered by a major challenge, which is tackling brain signal variability such as cross-session variability. Hence, with a view to develop good research practices in this field and to enable the whole community to join forces in working on cross-session estimation, we created the first passive brain-computer interface competition on cross-session workload estimation. This competition was part of the 3rd International Neuroergonomics conference. The data were electroencephalographic recordings acquired from 15 volunteers (6 females; average 25 y.o.) who performed 3 sessions—separated by 7 days—of the Multi-Attribute Task Battery-II (MATB-II) with 3 levels of difficulty per session (pseudo-randomized order). The data -training and testing sets—were made publicly available on Zenodo along with Matlab and Python toy code (https://doi.org/10.5281/zenodo.5055046). To this day, the database was downloaded more than 900 times (unique downloads of all version on the 10th of December 2021: 911). Eleven teams from 3 continents (31 participants) submitted their work. The best achieving processing pipelines included a Riemannian geometry-based method. Although better than the adjusted chance level (38% with an α at 0.05 for a 3-class classification problem), the results still remained under 60% of accuracy. These results clearly underline the real challenge that is cross-session estimation. Moreover, they confirmed once more the robustness and effectiveness of Riemannian methods for BCI. On the contrary, chance level results were obtained by one third of the methods—4 teams- based on Deep Learning. These methods have not demonstrated superior results in this contest compared to traditional methods, which may be due to severe overfitting. Yet this competition is the first step toward a joint effort to tackle BCI variability and to promote good research practices including reproducibility.

## 1. Introduction

Passive Brain-Computer Interfaces (BCIs) can estimate users' states, *e.g*., their cognitive or affective states, from their brain signals and use these estimations to adapt a human-computer interaction system accordingly (Zander and Kothe, 2011). As such, passive BCIs (pBCIs) have been used for many applications, including the estimation of users' mental workload (Roy and Frey, 2016), in order to adapt education material to students' cognitive resources (Yuksel et al., 2016) or to prevent airplane pilots from being overloaded (Singh et al., 2021a), and thus from missing alarms (Dehais et al., 2019a,b); or to estimate users' affective states, in order to design adaptive video games maximizing users' excitement or pleasure (Ewing et al., 2016), among many others.

As such, pBCIs are a key element for neuroergonomics (Dehais et al., 2020; Fairclough and Lotte, 2020a), for the design of numerous real-life studies and applications of neurotechnologies (Lotte and Roy, 2019). However, beyond promising proof-of-concepts, really using pBCIs in everyday life still requires to face numerous challenges. One of them is the well-documented large within-subject variability affecting brain signals such as ElectroEncephaloGraphy (EEG) signals. Indeed, EEG signals are highly non-stationary, and can change a lot across days, or within a day, even for the same user (Fairclough and Lotte, 2020b). However, so far, the vast majority of pBCI studies were conducted on a single day (*a.k.a*. session), making it unclear whether the designed BCI would still work on brain signals acquired over different days/sessions without re-calibration.

This is equally true for other EEG-based datasets and competitions outside the field of pBCI. Recent examples include the public database of joint recording of EEG and fNIRS data during cognitive tasks published by Shin et al. (2018) that enabled the authors to evaluate the benefits of a hybrid-BCI for within-subject and single-session mental state estimation during a word generation task. The *Clinical BCI challenge* in 2020 provided data from healthy and stroke patients, which was new and enabled both within and cross-subject estimation, but using only one session (consisting of 1 offline training session and 1 online testing session; Chowdhury and Andreu-Perez, 2021). Lastly, the *NeurIPS 2021 BEETL Competition: Benchmarks for EEG Transfer Learning* (Xiaoxi et al., 2021) provided 1 sleep dataset and 5 Motor-imagery datasets (active BCI). The goal was to perform cross-subject sleep stage estimation (impact of the age of the participants), as well as cross-dataset MI-BCI estimation with multiple sessions.

Hence, to our knowledge, existing public EEG datasets—and the scarce pBCI datasets—provide brain signals recorded on a single day/session (Hinss et al., 2021b), preventing the design and evaluation of pBCIs that would work across days/sessions. Ideally, a real-life pBCI, as envisioned in neuroergonomics, would need to be effective and efficient at all time, *i.e*., across sessions, without the need for recalibration. We thus proposed a data analysis competition at the 3rd International Neuroergonomics conference, that aimed at addressing this scientific challenge. We notably released the first (to the best of our knowledge) public pBCI dataset providing EEG signals across multiple sessions for each user, and we challenged the competitors to come up with the best algorithm to decode mental workload from EEG signals on a new unseen session, from a training dataset comprising several sessions. The choice was made to focus on a mental state frequently investigated in the pBCI literature, namely, mental/cognitive workload. Hence, data was acquired using a well-known task that elicits various levels of mental/cognitive workload: the Multi-Attribute Task Battery-II (MATB-II) developed by NASA, which enables to assess task-switching and mental workload capacities [https://matb.larc.nasa.gov—(Santiago-Espada et al., 2011)]. The dataset is part of a new open EEG database currently under development, and designed to address the need for more publicly available EEG-based datasets to design and benchmark passive brain-computer interface pipelines [as detailed in Hinss et al. (2021b)].

This article provides a retrospective on the competition by first detailing the competition management, the released dataset, the competitors and the methods employed by them as well as the obtained results and a reflection on which methods seem more fit for cross-session EEG-based mental workload estimation.

## 2. Competition & Dataset

### 2.1. Competition Management

The competition was organized as the grand challenge of the 3rd International Neuorergonomics conference (https://www.neuroergonomicsconference.um.ifi.lmu.de/) held online in September 2021 (from Munich, Germany). It was managed using the conference ConfTool submission website and the Zenodo dataset sharing website (Hinss et al., 2021a). The participation and submission rules were the following:

- One submission per team;
- One participant may only be part of one team;
- Submissions must include: results (estimated labels for the test set) and abstract (same format as regular paper submissions for the conference).

The important dates of the conference competition were the following:

- 15-Jun-2021: Official competition opening, publication of the (training) database (2 sessions with labels);
- 01-Jul-2021: Release of dataset version 2 with the 3rd session included as a test set (*i.e*., without labels);
- 31-Jul-2021: Competition closing, deadline for predictions and abstract submission;
- 31-Aug-2021: Evaluation by the competition organizing team;
- 05-Sep-2021: Submission of presentation materials;
- 13-Sep-2021: Announcement of results at the opening reception of the conference. Oral presentation of the winner and poster session presentations of all competing teams.

Overall, the goal of the participants was to design and to train a 3-class pBCI workload classifier on the first two labeled sessions—*i.e*., the training sessions—in order to predict the labels of the 3rd unlabeled session—*i.e*., the testing session. Participants should thus aim at reaching the highest classification accuracy on this testing session.

### 2.2. Participants and Protocol

The project was validated by the local ethical committee of the University of Toulouse (CER number 2021-342). Fifteen participants (6 females; average 25 y.o.) were invited to the lab for three independent experimental sessions, each spaced one week apart (exactly 7 days). Participants gave their written consent and received a monetary compensation (40 in total). Each session involved a short training/warm up period. Following this, a resting state (1 min with eyes open) was recorded. Participants then completed an MATB-II task with three 5-min blocks, each of a different difficulty level (*i.e*., different workload level) presented in a pseudorandom manner. By varying the number and complexity of the sub-tasks, 3 levels of workload were elicited (verified through statistical analyzes of both subjective and objective -behavioral and cardiac- data).

### 2.3. Task

**Figure 1:**
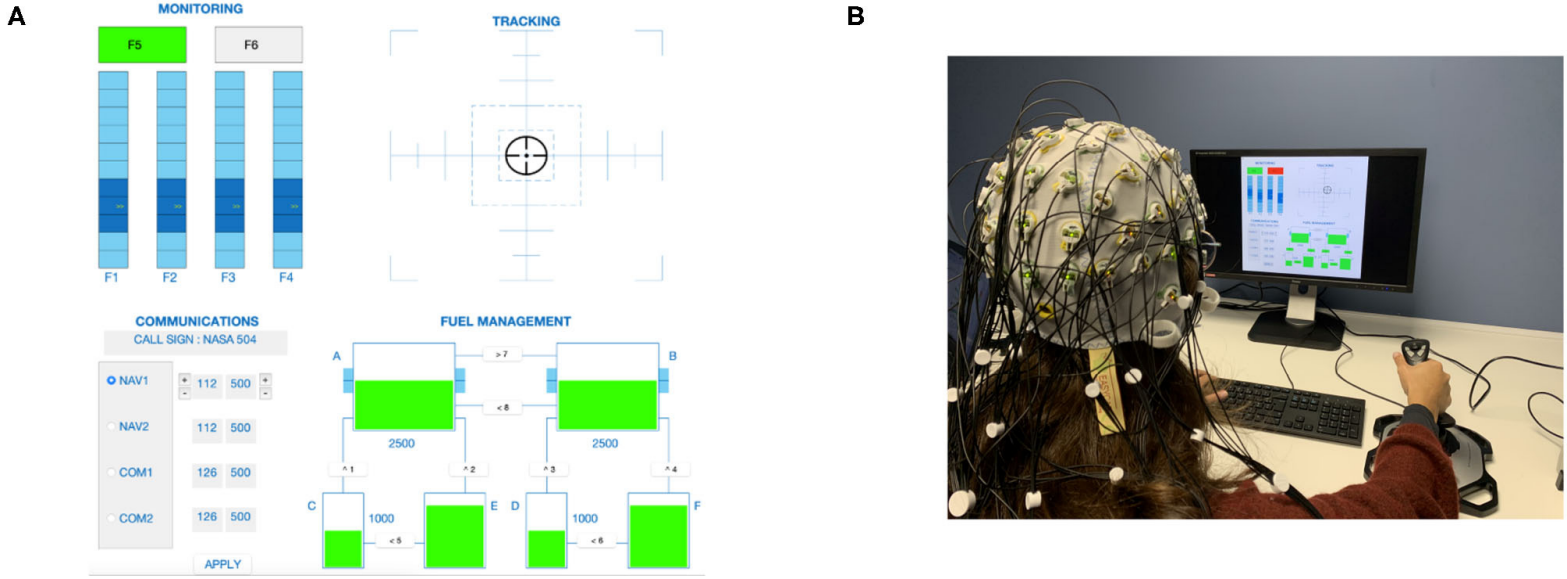
Experimental setup. **(A)** Screenshot of the MATB-II task performed by the participants, with four sub-tasks: namely (from the top left corner to the bottom right corner) Monitoring, Tracking, Communications, and Fuel management. **(B)** Database acquisition experimental setup.

As mentioned earlier, the dataset used for the competition comprised epoched data acquired during the performance of a well-known task in the human factors domain: the MATB-II whose graphical user interface is shown in Figure 1A. In this task, participants had to perform several sub-tasks simultaneously. Depending on the condition, the number of sub-tasks and their respective difficulty differed. Each condition lasted 5 min. The order was randomized with the other tasks, meaning that participants did not necessarily start with the easy task first.

In the easy condition (label 0), participants engaged in the TRACKING and SYSTEM MONITORING sub-tasks. The TRACKING task is a simulation of manual control, and the participant has to keep a target at the center of a window. The SYSTEM MONITORING task requires monitoring 4 gauges and 2 warning lights. For the medium condition (label 1), a third sub-task was added: RESOURCE MANAGEMENT. It presents the participant with a fuel management system where the goal is to maintain a certain fuel level by activating and deactivating a set of pumps that allow for the allocation of fuel to several reservoirs. Finally, for the difficult condition (label 2), the COMMUNICATION task was added to the three previous sub-tasks: here the participant has to respond to radio messages by changing the frequencies of different radios. Additionally, the TRACKING task was made more demanding in the DIFFICULT condition by increasing the speed of target motion.

### 2.4. Data Acquisition

A MATLAB version of the MATB-II task was used for the experimental campaign (developed and used by Verdiere et al. (2019); https://github.com/VrdrKv/MATB). EEG data acquisition was performed using a 64 active Ag-AgCl electrode system (ActiCap, Brain Products Gmbh) and an ActiCHamp amplifier (Brain Products, Gmbh; Figure 1B). One electrode could not be used and one electrode was dedicated to record cardiac activity, resulting in 62 electrodes, placed according to the international 10-20 system. In addition, the precise electrode location was obtained using a STRUCTURE (https://structure.io) 3D camera and the get_chanlocs plug-in developed specifically for electrode localisation purposes (https://github.com/sccn/get_chanlocs/wiki). The sampling frequency was set to 500 Hz. Impedance was kept below 10*kΩ* as much as possible. Data, as well as markers of all events occurring during the tasks, were recorded and synchronized using LabStreamingLayer (https://github.com/sccn/labstreaminglayer).

### 2.5. Data Preprocessing

Data preprocessing was done in MATLAB with the help of the EEGLAB toolbox (Delorme and Makeig, 2004). First, the data from the resting state as well as the tasks were extracted from the overall recording. The electrode recording cardiac activity was removed.

The following pre-processing pipeline was applied:

- Epoching into 2-s non-overlapping epochs;
- Referencing using right mastoid electrode;
- High-pass filter 1 Hz (FIR Filter, pop_filtnew from EEGLAB);
- Electrode rejection (average amplitude above 2 times the standard deviation across channels) and spherical interpolation;
- SOBI—a special case of blind source reconstruction based on second order statistics (Belouchrani et al., 1997)—with subsequent automated IC_Label rejection (muscle, heart, and eye components were rejected with a 95% threshold);
- Low-pass filter 40 Hz (FIR Filter);
- Average re-referencing (CAR);
- Down-sampling to 250 Hz.

Note that these preprocessing steps were performed in order to enhance the signal-to-noise ratio, but also to reduce biases in the competition. For instance, data were filtered below 40 Hz, and ICA was used so as to reduce as much as possible the risk of estimations based on motion-related ElectroOculoGraphy (EOG) and ElectroMyoGraphy (EMG) artifacts. These artifacts are mostly contained in the gamma band (Fatourechi et al., 2007). Hence, we wanted the competitors to base their method as much as possible on genuine cortical activity only. At the end of this preprocessing stage, for each of the different conditions and for each session there were 149 epochs extracted per participant.

### 2.6. Data Formatting

The data were exported as a dataset from EEGLAB under the *.set* and *.fdt* format. The COBIDAS BIDS formatting guidelines were followed (Pernet et al., 2020). Data were organized as follows:

- One directory per subject;
- Two sub-directories for each session;
- Inside each session directory one sub-directory for the precise electrode locations (measured *via* the STRUCTURE app) and one for the EEG data;
- 5 (*.set*) files per session. Each of the epoched and preprocessed task conditions, the resting state as well as the raw file for the resting state;
- Each epoch was marked by events to show the condition (difficult, medium, easy, Resting State);
- The electrode locations were provided in a. txt file with the xyz coordinates for each electrode.

## 3. Results

### 3.1. Competitors

**Figure 2:**
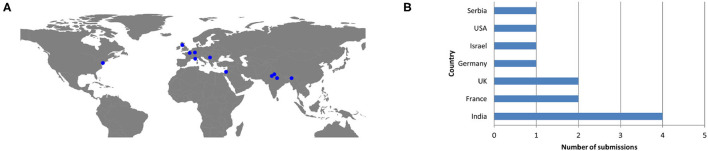
Teams' and submissions' geographical origin and number (first author). **(A)** Geographical origin of competition participants across the globe. **(B)** Number of submissions (and therefore teams) per country.

Eleven teams from 3 continents—Asia, Europe and North America (Figure 2A)—and 7 countries (Figure 2B), submitted their work, for a total of 31 participants. The biggest contributor in terms of submissions was India with 4 teams. France and the United Kingdom were behind with two teams (one was affiliated to both countries). Finally, Germany, Israel, Serbia, and the United States of America provided one submission each. The eleventh team was disqualified due to the submission of an incomplete results file.

### 3.2. Performances Obtained and Methods Used

The methods used by the contestants are listed and ranked with their achieved test classification accuracy in decreasing order in Table 1.

**Table 1:** Classification accuracy obtained, from best to worst, and methods used by the competition participants (winner score in bold).

**Team**	**Institution & Country**	**Test accuracy (3rd session)**	**Method**
Singh et al. (2021b)	IIT Kanpur and IIT Roorkee, India	**54.26%**	Riemannian geometry + automatic electrodes selection
Sedlar et al. (2021)	Inria, Sophia Anitpolis, France and Univ. Nottingham, UK	48.20%	Spherical CNN with rank-1 constraint
Corsi et al. (2021)	Inira, Paris, Univ. Paris-Saclay and Univ. Paris-Dauphine, France	48.13%	Riemannian geometry + functionnal connectivity features
Narayanan (2021)	Birl Institute of Technology and Science, Pilani, India	46.30%	Riemannian geometry
Bolton et al. (2021)	Tel Aviv University, Tel Aviv, Israel	44.67%	Random Forest on classical features (300)
Madhavan et al. (2021)	IIT Guwahati, Assam, India	43.79%	RNN on bandpower + approximate entropy features
Sharma (2021)	DFKI Saarbrcken, Germany	38.49%	Random Forest on CSP features
Kartali et al. (2021)	mBrainTrain LLC and Univ. of Belgrade, Serbia	33.18%	1 Dimension CNN
Kingphai and Moshfeghi (2021)	Univ. Strathclyde, Glasgow, Scotland	31.89%	RNN on frequency, statistical, morphological, time-frequency, linear, and non-linear features
De Lorenzo et al. (2021)	Drexel University, Philadelphia, USA	31.32%	RNN and CNN

In terms of classification accuracy, the obtained performances ranged from 31.32 to 54.26%. Note that the chance level for this 3-class problem is 33%, and an upper-bound of about 38% with the adjustment that takes the number of trials per class into account, following (Müller-Putz et al., 2008). Thus, 3 out of 10 valid submissions performed at chance level. The winner performed well above chance level, although with still quite a large error rate, the proposed classifier misclassifying almost every other epoch. This suggests that cross-session workload classification is a feasible but difficult task, for which there is still a lot of room for improvement.

In terms of methods, three main families of classifiers were explored: Riemannian geometry classifiers (Yger et al., 2016; Congedo et al., 2017) (in green in Table 1), deep learning classifiers (Roy et al., 2019) (in red in Table 1) and Random Forest classifiers (Breiman, 2001) applied onto classical features (in purple in Table 1). Roughly, Riemannian classifiers top the ranking, with the 1st, 3rd, and 4th best scores, classical approaches with Random Forests are in the middle of it (5th and 7th places), while the 3 worst performances, below chance level, are obtained by deep learning methods. A notable exception is the 2nd best performing approach, which uses a Convolutional Neural Network (CNN), *i.e*., a deep learning method.

Diverse standpoints on the data were exploited by these different classifiers. The first four teams from the ranking have taken advantage of some spatial information from the signal. Indeed, three methods used Riemannian geometry principles on covariance matrices extracted from the signals (Yger et al., 2016; Congedo et al., 2017). These covariance matrices encode spatial information. Corsi et al. (2021) have even added features from functional connectivity metrics. Sedlar et al. (2021) have used a specific type of CNN named spherical CNN, designed to perform convolutions on non-planar data as the layout of electrodes on the skull (planar data are for example 2D images). It thus specifically exploited the topographical layout of EEG electrodes, *i.e*., domain-specific prior knowledge.

The winning solution from Singh et al. (2021b) has also taken advantage of an automatic and per-subject electrode selection to reduce the number of electrodes from 61 to 18−32, in addition to a classical approach using Riemannian geometry principles to project covariance matrices to the tangent space and use these projections as features to feed a Support Vector Machine (SVM) classifier. The selection was made sequentially, by pruning channels, to find the combination that maximises the Riemannian distance between class-conditional covariance matrices.

**Figure 3:**
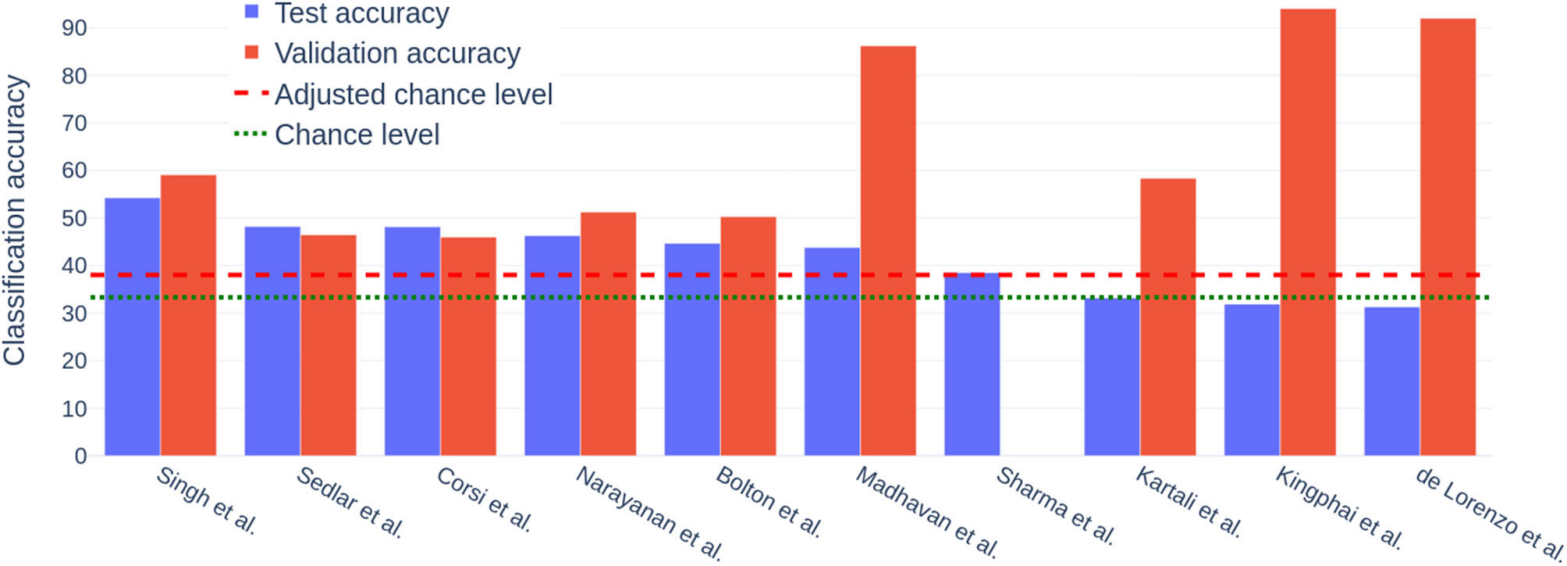
Difference between the test accuracy (blue; from sessions 1 + 2 to 3), computed by the organizers, and the validation accuracy (orange; from sessions 1 to 2 or reverse), computed locally by competitors. A large discrepancy between the two indicates an issue with a generalization, *i.e*., over-fitting.

In Figure 3, the discrepancy of the validation accuracy (evaluated by the competitors using the published dataset—sessions 1 and 2) and the test accuracy (evaluated by the organizers using session 3) is depicted. Validation accuracy from Sharma (2021) was missing in the report and is therefore omitted from the graph. Teams are ordered from left to right following the ranking of the competition. For the first five competitors, the overfitting seems limited as the validation accuracy are close to test accuracy. However, for the three deep learning methods at the end of the ranking, generalization seems to have been an issue as the methods perform very well from session 1 to 2 but at chance level on session 3. All deep learning models, except the one from Sedlar et al. (2021), have proposed quite large models, thus with many parameters, and have used Recurrent Neural Network (RNN) models—except Kartali et al. (2021) who used CNN, which may explain the generalization issues. Indeed, it could be that there was not enough training data to properly train such large deep learning methods, with many parameters and little prior knowledge. This seems to be confirmed by the performances obtained by the 2nd best performing method, by Sedlar et al. (2021), as it also uses a deep learning algorithm, however, with few parameters and layers (it was actually quite a shallow neural network) and with strong prior about EEG generation.

Interestingly, it can be noticed that none of the methods proposed actually performed explicit inter-session transfer using, e.g., transfer learning (Jayaram et al., 2016; Azab et al., 2018). Indeed, the participants tried to design models with the higher generalization power, so that by training it on session 1 and 2 it would still perform well on session 3. No statistics or characteristics were extracted from session 3 to try to adjust/transfer the predictions. Similarly, no information about other subjects were used to train the classifiers for a given subject. In other words, neither cross-session nor cross-subject transfer was explored. Several participants have mentioned this transfer learning approach as future work in their abstract and therefore it seems to remain an open question.

## 4. Conclusion

Overall, in this article, we described our efforts toward moving pBCI technologies beyond proof-of-concept studies in a single session, to more realistic pBCI use across multiple days/sessions, *i.e*., in a neuroergonomic approach. In particular, we organized the first pBCI competition that aimed at estimating workload levels (with three levels of workloads) across sessions, with two sessions for training the BCI classifier, and one session for testing it. For that competition, we collected a dedicated EEG data set, that we publicly shared with the community—in order to stimulate research in that direction even beyond the competition.

The results of that competition provided several interesting insights. First, it confirmed the effectiveness and superiority of Riemannian geometry classifiers for BCI, whether active or passive, as the winner used Riemannian classifiers, and 3 out of the 4 best scores were obtained using Riemannian geometry. However, this claim should be tempered by the fact that the number of participating teams was only of 10 and that achieved accuracy is quite low. Besides, we could also notice that a classical approach here submitted by Bolton et al. (2021), with a traditional feature extraction and a random forest as classifier has achieved comparable results to those obtained with the other methods of the top-5.

The overfitting of 3 out of 4 of the deep learning methods highlights that deep learning is not a silver bullet. It requires some careful design of an adapted architecture and training procedure to the dataset. Here, as in all BCI research so far, the dataset was small. Hence, overfitting due to too large networks was a clear pitfall. A notable exception, the 2nd best results, used a neural network method with a compact (shallow) network and strong prior knowledge about EEG generation. Thus, deep learning seems useful for BCI when it is not deep, and does not fully apply end-to-end learning, but rather (manually) integrate prior knowledge in its architecture. The difficulty of designing an adequate deep learning method for BCI was also observed elsewhere [see, e.g., (Lotte et al., 2018) for a review]. Moreover, even when properly applied, the deep learning methods applied to BCI do not offer an edge over other traditional methods, like it has revolutionized computer vision or natural language processing. Hence, it seems that there is no deep learning revolution in BCI, at least so far.

Finally, the results obtained by the competitors, in terms of classification accuracy, revealed that while cross-session workload classification is feasible, the robustness achieved is still rather low, and would require a lot of improvement for being used in practical applications outside the lab. Yet, it should be noted that the database provided for this competition could have been larger. Indeed, at the time of the competition opening only 15 participants had undergone the 3 acquisition sessions, with an unbalanced database in terms of gender. This was mainly due to participant recruitment and data acquisition constraints in times of covid-19 pandemic, and will need to be further addressed with the release of a more complete and richer database.

Nevertheless, such results thereby open interesting perspectives for future research. First, they stress the need for more BCI studies across sessions, both to assess existing pBCIs (usually designed on a single session) but also to design new algorithms able to deal with cross-session variabilities. To do so, transfer learning algorithms (across sessions or users) seem to be promising approaches to explore, that the competitors of that competition have not employed yet. It should also be stressed that cross session variabilities are only a single type of variabilities that affect BCI performances. Many other sources of variabilities affect BCIs, such as cross-subject, cross-context, cross-task or change in users' mental states, among other (Roy et al., 2013; Fairclough and Lotte, 2020b). Thus, we hope that such a competition highlighted the need for more studies, algorithm designs, benchmarks or data base collections to tackle variabilities in BCI in general.

## Data Availability Statement

The datasets presented in this study can be found in online repositories. The names of the repository/repositories and accession number(s) can be found at: https://doi.org/10.5281/zenodo.5055046.

## Ethics Statement

The studies involving human participants were reviewed and approved by Ethical Committee of the University of Toulouse (CER number 2021-342). The patients/participants provided their written informed consent to participate in this study.

## Author Contributions

RR, LD, and FL: drafting of this article. MH, SL, XX, ND, and FD: critical revisions. MH and RR: database design. MH, EJ, and BS: database acquisition. LD, MH, and SL: result analysis. RR, MH, LD, and SL: competition management. RR: supervision. All authors contributed to the article and approved the submitted version.

## Funding

This work was funded by ANITI (Artificial and Natural Intelligence Toulouse Institute), Toulouse, France. FL was also supported by the European Research Council with project BrainConquest (grant ERC-2016-STG-714567).

## Conflict of Interest

The authors declare that the research was conducted in the absence of any commercial or financial relationships that could be construed as a potential conflict of interest.

## Publisher's Note

All claims expressed in this article are solely those of the authors and do not necessarily represent those of their affiliated organizations, or those of the publisher, the editors and the reviewers. Any product that may be evaluated in this article, or claim that may be made by its manufacturer, is not guaranteed or endorsed by the publisher.
